# Maize/soybean intercropping with nitrogen supply levels increases maize yield and nitrogen uptake by influencing the rhizosphere bacterial diversity of soil

**DOI:** 10.3389/fpls.2024.1437631

**Published:** 2024-09-03

**Authors:** Liqiang Zhang, Yudi Feng, Zehang Zhao, Zhengguo Cui, Bate Baoyin, Hongyu Wang, Qiuzhu Li, Jinhu Cui

**Affiliations:** ^1^ College of Plant Science, Jilin University, Changchun, China; ^2^ Soybean Research Institute, Jilin Academy of Agricultural Sciences, Changchun, China

**Keywords:** maize-soybean intercropping, microbial community, nitrogen fertilizer use, soil nitrogen, yield

## Abstract

**Introduction:**

Intercropping practices play a crucial role in enhancing and maintaining the biodiversity and resiliency of agroecosystems, as well as promoting stable and high crop yields. Yet the relationships between soil nitrogen, microbes, and yield in maize cultivated under maize/soybean intercropping systems remain unclear.

**Methods:**

To fill that knowledge gap, here we collected maize rhizosphere soil at the staminate stage after 6 consecutive years of maize/soybean intercropping, to investigate how intercropping and nitrogen application rates affected nitrogen utilization by crops and soil microbial community composition and function. We also examined correlations of those responses with yields, to clarify the main ways that yield is enhanced via intercropping and by nitrogenous fertilizer gradient changes generated by different nitrogen application rates.

**Results:**

The amount of applied fertilizer was 240 kg N ha^-1^ was best for obtaining a high maize yield and also led to the greatest nitrogen-use efficiency and bacterial diversity. Under the same N application rate, intercropping increased the maize yield by 31.17% and soil nitrogen (total, ammonium and nitrate nitrogen) by 14.53%, on average, in comparison to monocropping. The enrichment of *Gemmatimonas* and *Bradyrhizobium* significantly increased the soil nitrogen content, and a greater relative abundance of *Sphingomonas* and *Gemmatimonas* increased the maize yield, whereas enrichment of *Candidatus_Udaeobacter* and *Bradyrhizobium* decreased it. The benefits of intercropping mainly arise from augmenting the abundance of beneficial microorganisms and enhancing the efficiency of N use by crop plants.

**Discussion:**

This study’s findings are of key importance to bolster the stability of agro-ecosystems, to guide the scientific rational use of nitrogen fertilizers, and to provide a sound theoretical basis for achieving the optimal management of intensive crop-planting patterns and green sustainable development.

## 1 Introduction

As one of the world’s staple food crops, maize figures prominently in global agricultural production (Zhou and Butterbach-Bahl, 2014). However, the continuous cultivation of a single crop—i.e., monocropping or a monoculture system—can lead to declining soil quality and its greater susceptibility to pests and diseases (Du et al., 2018). Hence, it is imperative that farmers begin to scientifically and rationally apply maize/soybean intercropping planting techniques to improve their crop yields and economic benefits. Intercropping is a type of ecological agriculture that improves the accumulation of nutrients in soil for uptake by plants, to promote their growth via complementary trait advantages among different crop species (Fan et al., 2023). Proper intercropping patterns can bolster soil structure, alter soil physicochemical properties, and increase soil’s nutrient contents and enzymatic activity (Secco et al., 2023). Improved soil conditions then enhance plants’ growth, physiology, and nutrient uptake, leading to higher biodiversity overall, which also entails a greater overall yield of intercropping systems (Yang et al., 2020). Intercropping also enriches the diversity of soil microbial communities, improves soil health, and helps to balance beneficial microorganisms vis-à-vis harmful ones, all of which is advantageous for plant growth and development (Guo et al., 2024).

Nitrogen (N) is the most basic element of life, being a key component of proteins, nucleic acids. and other essential substances of living organisms, and thus directly involved in myriad biological processes (Bai et al., 2023). Regarding N uptake and N use in grass-bean intercropping systems, the proven benefits of maize/soybean intercropping are known to include an increased effective N content of soil and fixing of N and its transfer to grasses, thus reducing their demand for chemical N fertilizers and improving the efficiency of water use and other nutrient uses (Lu et al., 2023). In recent years, whether the N fixed by legumes can be transferred to adjacent grass crops in intercropping systems has emerged as a hot research topic (Chen et al., 2023; Liu et al., 2023). Further, intercropping with cereals can reduce the “N-deterrent” effect of chemical N fertilizers on legumes, thereby promoting legume nodulation and increasing the rate of N fixation (Xu et al., 2020). Overall, the two paramount reasons for efficient N use in grass/legume intercropping systems are “N transfer” and “N repression”, with both phenomena essentially the outcome of N competition dynamics and complementarity between grass crops and legumes (Zhang et al., 2017).

A healthy soil environment underpins high and stable crop yields and is shaped by land use and land nutrition (Qi et al., 2023). One study showed that rhizosphere soil pH fell markedly in a soybean monoculture, but intercropping was able to mitigate that decline (Zaeem et al., 2019). However, intercropping has a dual effect on the organic matter content of soil. On one hand, intercropping can increase it by enhancing ecological interactions in the soil microenvironment that hasten the decomposition of plant and animal residues (Zhu et al., 2024); on the other hand, plants compete for soil nutrients, which accelerates mineralization, the decomposition of soil organic matter, and nutrient cycling, collectively reducing soil’s organic matter content (Roohi et al., 2022).

In intercropping systems, competition and facilitation effects that arise between plant species often enrich the diversity of soil microbial communities and enlarge microbial populations, culminating in mutual benefits and co-promotion achieved under appropriate intercropping modes (Zhang et al., 2023). Previous research has found a substantially increased number of soil bacteria and actinomycetes, but a considerable decrease in the number of fungi under maize/soybean intercropping vis-à-vis maize monocropping (Fu et al., 2019). According to work by Tian et al. (2019), a greater relative abundance of soil beneficial bacteria belonging to dominant taxa, such as Proteobacteria and Acidobacteria, and likewise dominant fungi, namely Ascomycota and Basidiomycota, can help to augment the overall number of beneficial microbes in soil. Recently, Li et al. (2021) showed that the structure of soil microbial populations was altered in intercropped peanut and cereal crops, resulting in less bacteria that are harmful (such as *Spiroplasma*) and more bacteria that are beneficial (such as Streptomycetaceae and *Bacillus*). The above studies uncovered impacts of intercropping for improving soil microbial population types and community structure.

While those studies do provide a good summary of recent progress towards elucidating the effects of intercropping systems upon specific soil environments, the experimental methods employed tend to differ with intercropping patterns varying widely. In recent years, research on maize/soybean intercropping has focused chiefly on the relationship between light energy use and crop yield (Yao et al., 2017; Zheng et al., 2022), leaving far less known about how maize soil N use in combination with changes in the rhizosphere soil microbial community could affect their maize yield. Here we sampled maize soils at the male withdrawal stage in a continuous 6-year maize/soybean intercrop system, then quantified the use of soil nitrogen in its transformation and determined the bacterial community composition via high-throughput sequencing. This study had three objectives: (1) to quantify the effect of maize/soybean intercropping on maize soil nitrogen-use efficiency; (2) to distinguish the influential factors and mechanisms linking the maize-soybean intercropping system to soil microbial changes in the maize rhizosphere; and (3) to clarify the interactions between the soil microenvironment and maize yield under a maize/soybean intercropping system. This study provides a theoretical basis for the implementation of optimal management and environmentally sustainable development of intensive cropping systems.

## 2 Materials and methods

### 2.1 Study area

The experiment was run at the Agricultural Experimental Base of Jilin University, Changchun City, Jilin Province (125°14.23*’* E, 43°56.60*’* N; elevation: 245 m a.s.l). The site has a black soil type, a temperate continental semi-moist monsoon climate, with an average annual precipitation of 600–700 mm and average annual temperature of 4.6°C (max: 40°C and min: –36.5°C). The annual frost-free period lasts 140–150 days and the annual freezing period spans 150–160 days. The soil type in the field is Phaeozems (FAO-WRB classification system, 2014).Prior to the experiment, the pH value in the soil was 5.33, and it had these characteristics: organic carbon (1.37%), total nitrogen (1.41 g·kg^-1^); total phosphorus (0.48 g·kg^-1^) and total potassium (21.42 g·kg^-1^).

### 2.2 Experimental design

**Figure 1:**
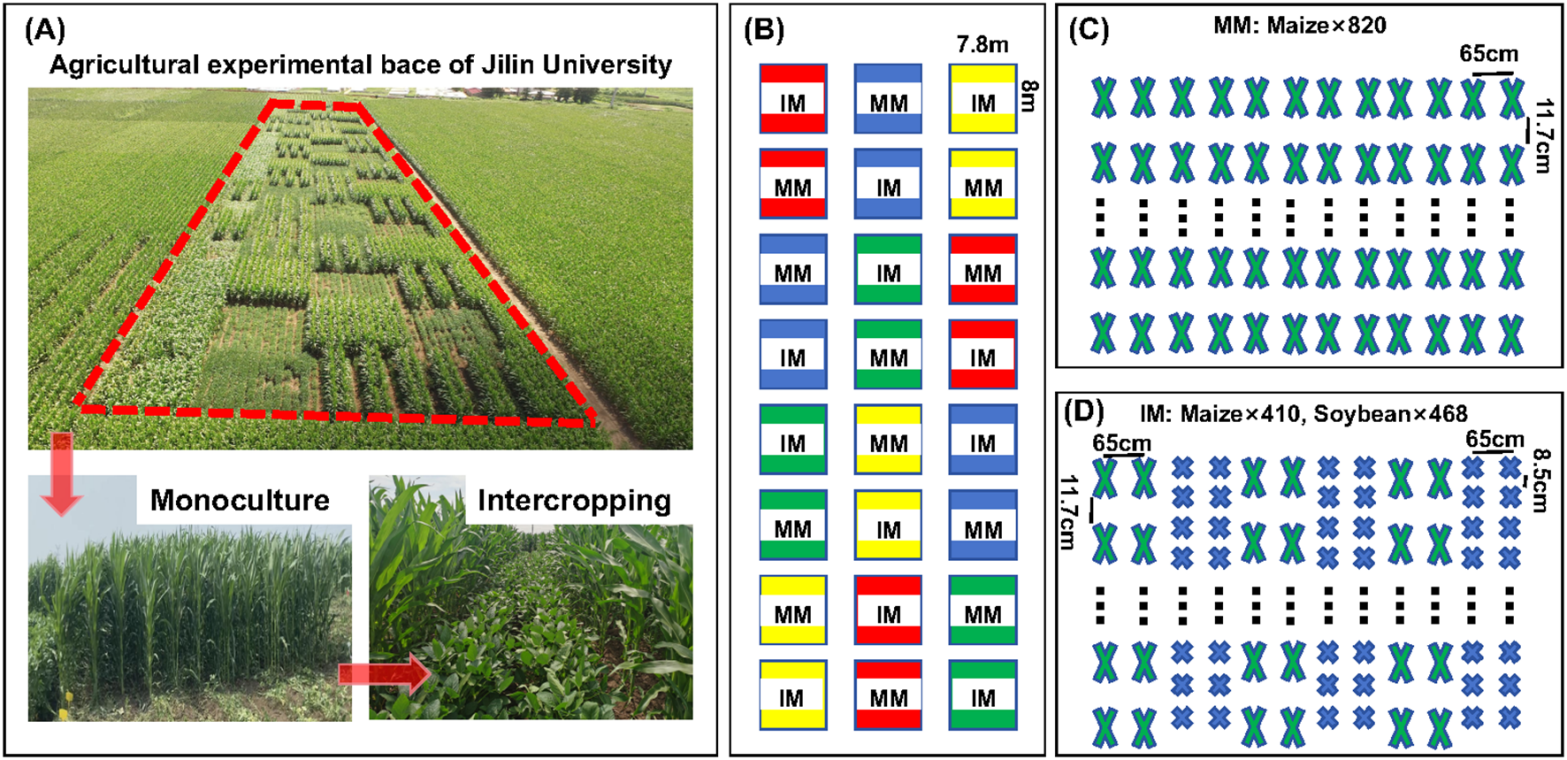
Aerial view **(A)** and schematic **(B)** layout of the maize/soybean intercropping experiment under different nitrogen application rates. In (B), red is the N0 level, blue is the N1 level, green is the N2 level, and yellow is the N3 level. **(C)** distribution of maize monoculture (MM) trial plots; **(D)** distribution of maize–soybean intercropping (IM) trial plots. where 820, 468, and 410 are the number of crop plants in the plot; 65 cm refers to the spacing between rows, while 8.5 cm or 11.7 cm is the spacing between plants within a given row.

This trial was established as a long-term locational trial, begun 2017. Its fertilizer application rates, and tillage practices remained consistent across all treatments from 2017 through 2023. Likewise, the same field management practices were maintained across the treatments during the fertility period in every year. While the experiment was continued from 2017 to 2023, the samples for analyses were taken in 2023 growing period. This experiment used a two-factor split-plot design, with the nitrogen supply level assigned to the main plots (blocks) and the planting mode to the subplots (strips). The maize variety used for testing was ‘Xianyu 335’ and the soybean variety was ‘Changnong 16’, both supplied by the Jilin Academy of Agricultural Sciences. Their seeds were sown on 26 April 2023, with the maize monoculture (MM) serving as the control group and intercropping of maize and soybean (IM) (maize/soybean, 2:2) set as the experimental group. Each plot was 62.4 m^2^ in size and contained 12 rows of maize in the monoculture plot. The maize/soybean intercropping plot consisted of three strips: each strip was planted with two rows of maize and two rows of soybean. The row spacing was 65 cm and the planting density was 90 000 plants ha^-1^ for maize and 180 000 plants ha^-1^ for soybean (**Figure 1**). Fertilizer application levels were the same for the intercropping and monocropping plots, with maize receiving 0 (N0), 180 (N1), 240 (N2), or 300 (N3) kg N ha^-1^. Basal fertilizer rates were 0, 40, 80, and 120 kg N ha^-1^, with the remainder of the nitrogen fertilizer applied as a top-up fertilizer at the nodulation stage of maize. The phosphorus fertilizer consisted of heavy calcium superphosphate (P_2_O_5_ 46%) applied at 120 kg P_2_O_5_ ha^-1^; the potassium fertilizer was potassium sulfate (K_2_O 50%), it applied at 100 kg K_2_O ha^-1^. Both phosphorus and potassium fertilizers were applied as basal applications.

### 2.3 Soil and plant sampling

Surface soil (0–20 cm depth, between adjacent maize on the ridge) and rhizosphere soil samples were collected on 21 July 2023 (at the maize staminate stage). Surface soil was sampled using a soil auger, at five points selected from each plot (in an X formation), with root samples were taken by drilling *in situ* on the ridge using an Eikelkamp root auger (auger diameter 8.0 cm, length 20.0 cm, 75% ethanol disinfection). After air-drying the soil samples indoors and passing them through a 75-mesh sieve, the ensuing fine-grained soil was used for the determination of soil chemical properties. To collect rhizosphere soil, three consecutive plants were excavated from each plot in the field, with their roots and attached soil intact. The top layer of soil was then removed; then, soil loosely bound to the root system was gently shaken and collected, and this mixed it together as a composite sample of rhizosphere soil for later testing (n = 6). All these rhizosphere soil samples were stored in a freezer at –80°C until their microbial diversity analysis. When the maize plants were mature, two rows from each plot were harvested; after threshing all their kernels, the grain weight was measured and converted to hectare yield based on the area harvested. The kernels’ moisture content was recorded with a PM8188 moisture meter, for three replicates, and the final yield data then expressed as the seed (grain) weight at 14% moisture. Panicle length, ear diameter, number of grains, 100-seed weight, and barren tip size were recorded as well.

### 2.4 Soil and plant sampling

#### 2.4.1 Soil nutrients

To determine soil pH and EC (electrical conductivity), a water-to-soil ratio of 5:1 was used, this shaken at 180 r/min for 5 min and allowed to stand for 30 min before taking the respective measurements with a pH meter (pH-100A,100-2000 rpm, LICHEN, Shanghai, China) and a conductivity meter (DDSJ-11A-307, YUEPING, Shanghai, China). Soil ammonium nitrogen (NH4^+^-N) and nitrate nitrogen (NO_3_^–^N) fractions were extracted with sodium bicarbonate, shaken at 180 r/min for 2 h, allowed to stand for 30 min, and then filtered through a 0.45-μm PES membrane. For total soil nitrogen (TN), samples were treated with Kjeldahl digestion and filtered through a 0.45-μm PES membrane, and then measured by a continuous flow analyzer (AA3, AutoAnalyzer 3, Technician, Windows/NT) (Wang et al., 2023a). Soil total organic carbon (TOC) fractions were determined by wrapping 10-mg soil samples in aluminum foil and using an elemental analyzer (vario TOC cube, NDIR, 60 000 ppm, 2 ppb, GER) (Zhu et al., 2021).

#### 2.4.2 Nitrogen utilization

Just before the field was harvested, five intact maize plants with uniform and typical growth were selected from each plot, and both parts oven-dried at 105°C for 30 min and then again, at 75°C, to a constant weight. After weighing the dry matter, the samples were crushed, digested with H_2_SO_4_-H_2_O_2_, and the nitrogen content of the maize plants and kernels determined using a Seal AA3 flow analyzer. Nitrogen-use efficiency (NUE) (i), nitrogen agronomic efficiency (NAE) (ii), nitrogen partial productivity (NPP) (iii) and nitrogen harvest index (NHI) (iv) were calculated as follows (Bai et al., 2024):

- NUE (%) = (N accumulation of aboveground plants in the N-applied area – N accumulation of above-ground plants in non-N applied area)/N application × 100.
- NAE (kg·kg^-1^) = (seed yield in the N-applied area – seed yield in non-N applied area)/nitrogen application rate × 100.
- NPP (kg·kg^-1^) = seed yield/applied nitrogen.
- NHI (%) = total seed N accumulation/total plant N accumulation.

#### 2.4.3 Diversity of soil bacterial communities

Total DNA was extracted from each soil sample using a DNA kit (MN NudeoSpin 96 Soi) and the DNA concentration was then determined on a NanoDrop 2000 spectrophotometer. The PCR reaction conditions were as follows: pre-denaturation at 94°C for 5 min and then 30 cycles at 94°C for 30 s, 50°C for 30 s and 72°C for 60 s, followed by a stable extension at 72°C for 7 min, and finally stored at 4°C. The extracted genomic DNA was detected by 1% agarose gel electrophoresis. For the bacterial 16S gene, the primer pair of 338F (5’-ACTCCTACGGGAGGCAGCAG-3’) and 806R (5’-GGACTACHVGGGTWTCTAAT-3’) was used to amplify the V3-V4 region, and the ensuing products were purified, quantified, and standardized. Information about the hardware and software used for sequencing is presented in **Table 1**.

**Table 1:** Information on the sequencing instruments and reagents used in this study.

Types	Instruments/Reagents	Producers	Specification/Model/Lot Number
Amplicon extraction	MoBio PowerSoil DNA Isolation Kit (100)	QIAGEN	100 times
Amplifier amplification	KAPA 2G Robust Hot Start Ready Mix	KAPA	
	ABI 9700 PCR	ABI	
Amplicon purification	Agencourt^®^ AMPure^®^ XP	Beckman Coulter	Dispense 45 mL/bottle, total 450 mL/bottle
Amplicon building	NEBNext Ultra II DNA Library Prep Kit	NEB	96 reactions
	Agencourt^®^ AMPure^®^ XP	Beckman Coulter	Dispense 45 mL/bottle, total 450 mL/bottle
	ABI 9700 PCR	ABI	
Library quality control instruments	Bioanalyzer (Agilent 2100)	Agilent	DE13806339
	Biomolecule Analyzer (Labchip GX)	PerkinElmer	
	ABI Qpcr	ABI	Step One Plus
Library quality control reagents	Agilent DNA 1000 Kit	Agilent	300 samples
	HT DNA-Extended Range LabChip	PerkinElmer	
	KAPA Library Quantification Kit	KAPA	500 times
Sequencing equipment	High-throughput second-generation sequencer	Illumina	MiSeq
Sequencing reagents	MiSeq^®^ Reagent Kit v3 (600 cycle) (PE300)	Illumina	
	MiSeq Reagent Kit v2 (500 cycle)	Illumina	

Library construction went as follows: (1) ligation of the ‘Y’ junction; (2) removal of junction self-associated fragments by magnetic bead screening; (3) enrichment of the library template via PCR amplification; (4) denaturation by sodium hydroxide to produce single-stranded DNA fragments.

Next, these eight sequencing steps were applied: (1) one end of the DNA fragment that is complementary to the primer base is fixed on the chip; (2) its other end randomly complementary to another primer in the vicinity is also fixed, to form a ‘bridge’; (3) PCR amplification is carried out to produce DNA clusters; (4) the DNA amplicon is linearized into a single strand; (5) add the modified DNA polymerase and dNTP with four fluorescent labels, synthesizing only one base per cycle; then (6) scan the surface of the reaction plate with a laser to read the nucleotide species polymerized in the first reaction of each template sequence; (7) chemically cleave the “fluorescent group” and the “termination group” to restore the 3’-end attachment, and continue to polymerize the second nucleotide; and end by (8) counting the fluorescence signals collected in each round to obtain the sequence of the template DNA fragment (Wang et al., 2022b). All obtained sequences were deposited, and expression data uploaded to the NCBI (National Center for Biotechnology Information) (https://www.ncbi.nlm.nih.gov/) (accessed on 13 June 2024), under BioProject ID: PRJNA1132549.

### 2.5 Soil and plant sampling

Statistical analyses were implemented using SPSS 22.0 software to compare the effects of intercropping upon the soil response variables, using univariate two-way ANOVAs. Pearson correlations were used to evaluate the relationships between relative abundance of dominant genera, soil chemical properties, and maize yield. Beta diversity analysis was done based on the coefficient of variation of the Aitchison distance, and principal component analysis (PCA) used to compare the degree of similarity present between different samples in terms of species community diversity. The visual analysis of microbial ecological networks and derivation of topological indices were carried out in Gephi software (v0.9.6). The following topological indices were used to describe the nodes and connecting lines in the constructed microbial network: (1) the number of connecting lines of a node, which is the sum of all lines linked to each node; (2) the median centrality of a node, which is the node located on the shortest path between two nodes, this calculated according to the Formula 1 below; (3) the topological coefficient of a node, which conveys the proximity of nodes and is expressed by Formula 2 below; (4) the connecting line weight, which reflects the number of connections between a particular OTU (operation taxonomic unit) node and the sample node; (5) the connecting line centrality, a parameter that gauges the importance of each connecting line in the information transfer process of the whole network (Faust, 2021). Structural equation modeling (SEM) of the direct or indirect effects of intercropping and nitrogen application rates on yield pathways was implemented using R v4.3.1 (https://www.r-project.org/).


(1) $$\begin{array}{c} Cb\left(n\right)=\sum_{s\neq n\neq t}\left(\frac{\sigma_{st}\left(n\right)}{\sigma_{st}}\right) \end{array}$$

In the above equation, *n* is the destination node; *s* and *t* are nodes in the network other than *n*; $\sigma_{st}$ denotes the number of shortest paths from node *s* to node *t*, and the term $\sigma_{st}\left(n\right)$ denotes the number of shortest paths from node *s* to node *t* that must pass through node *n*.


(2) $$\begin{array}{c} T_{n}=\frac{avgJ_{\left(n,m\right)}}{k_{n}} \end{array}$$

Here, $J_{\left(n,m\right)}$ is the number of all nodes adjacent to both nodes *n* and *m*, where the value of $J_{\left(n,m\right)}$ is increased by 1 if *n* is directly adjacent to *m*; $k_{n}$ is the number of all neighbours of that node.

## 3 Results

### 3.1 Maize yield

**Table 2** presents the effects from the two contrasting modes of cropping and from different nitrogen application rates on the maize yield factors. When their comparing maize yields under the same nitrogen application rates, in all cases IM (intercropping maize) > MM (monoculture maize), with maize yields at N0, N1, N2, and N3 fertilizer levels increased by 45.39%, 25.57%, 25.15% and 28.58%, respectively. From a nitrogen application perspective, the strongest yield effect occurred in going from the N0 to N1 level with an increase of 53.77% and 32.49% in MM and IM, respectively. That increase fell correspondingly to 12.31% and 12.21% at the N2 level; however, when a greater nitrogen application was used, i.e., N3 level, the maize yield decreased by 8.27% and 5.76% relative to that obtained for N2.

**Table 2:** Yields and yield factors of maize in response to two contrasting cropping systems (C) and four nitrogen application rates (N1–4).

Treatment		Panicle length (cm)	Barren tip (cm)	Ear diameter (cm)	Grain number	100-seed weight (g)	Yield (kg·ha^-1^)
MM	N0	14.92 ± 0.86c	2.06 ± 0.22a	4.69 ± 0.04c	405 ± 53.7d	35.24 ± 1.25d	8897 ± 602e
	N1	18.21 ± 0.53b	1.63 ± 0.34ab	4.90 ± 0.03b	575 ± 60.3bc	38.54 ± 1.04bc	13681 ± 716d
	N2	17.94 ± 0.64b	1.60 ± 0.67ab	4.79 ± 0.11bc	596 ± 79.7b	35.46 ± 0.33cd	15365 ± 494c
	N3	17.95 ± 0.43b	1.49 ± 0.49ab	4.91 ± 0.14bc	594 ± 55.9b	36.88 ± 2.20cd	14095 ± 911cd
IM	N0	17.53 ± 0.43b	1.50 ± 0.61ab	4.85 ± 0.06b	547 ± 59.5c	36.13 ± 2.03cd	12935 ± 302d
	N1	19.39 ± 0.15a	0.98 ± 0.26b	4.95 ± 0.26ab	601 ± 39.8b	41.58 ± 2.55ab	17138 ± 794b
	N2	19.97 ± 0.51a	0.86 ± 0.12b	5.13 ± 0.12a	649 ± 62.2a	41.73 ± 2.65a	19230 ± 386a
	N3	19.75 ± 0.27a	0.91 ± 0.58b	5.11 ± 0.58a	637 ± 90.0a	42.52 ± 1.46a	18123 ± 786ab
Two-factor variance analysis (*F*-value)							
	N	**	**	**	**	**	**
	C	**	*	**	**	**	**
	N*C	ns	ns	ns	ns	*	ns

Different lowercase letters (a, b, c) indicate significant differences between treatments (P < 0.05). Asterisks indicate a significant effect on the response variable: * P < 0.05, ** P < 0.01. MM, monoculture; IM, intercropping. ns indicate that there is no significant effect on the response variable.

The effects on panicle length, ear diameter, grain number, and 100-seed weight were consistent with the maize yield changes, in that all four were higher under IM than MM, but vice versa for barren tip (MM > IM). Most of the yield factors in the MM treatments showed this response, of N1 > N2, with the exception of only a lower grain number and longer barren tip for the N2; hence, the N1 yield under MM was lower than that under N2 mainly due to the effects on grain number and barren tip, while all yield factors in the IM treatment peaked at the N2 level.

The two-factor ANOVAs showed that the nitrogen application rate (N) as well as cropping mode (C) significantly affected all the indicators (P < 0.01), whereas their interaction effect (C*N) was only significant for the 100-seed weight (P < 0.05). Evidently, the N2 treatment was the better level of N application irrespective of planting mode (MM and IM), and IM coupled with N2 resulted in the highest maize yield.

### 3.2 Soil chemistry and nitrogen content

**Figure 2:**
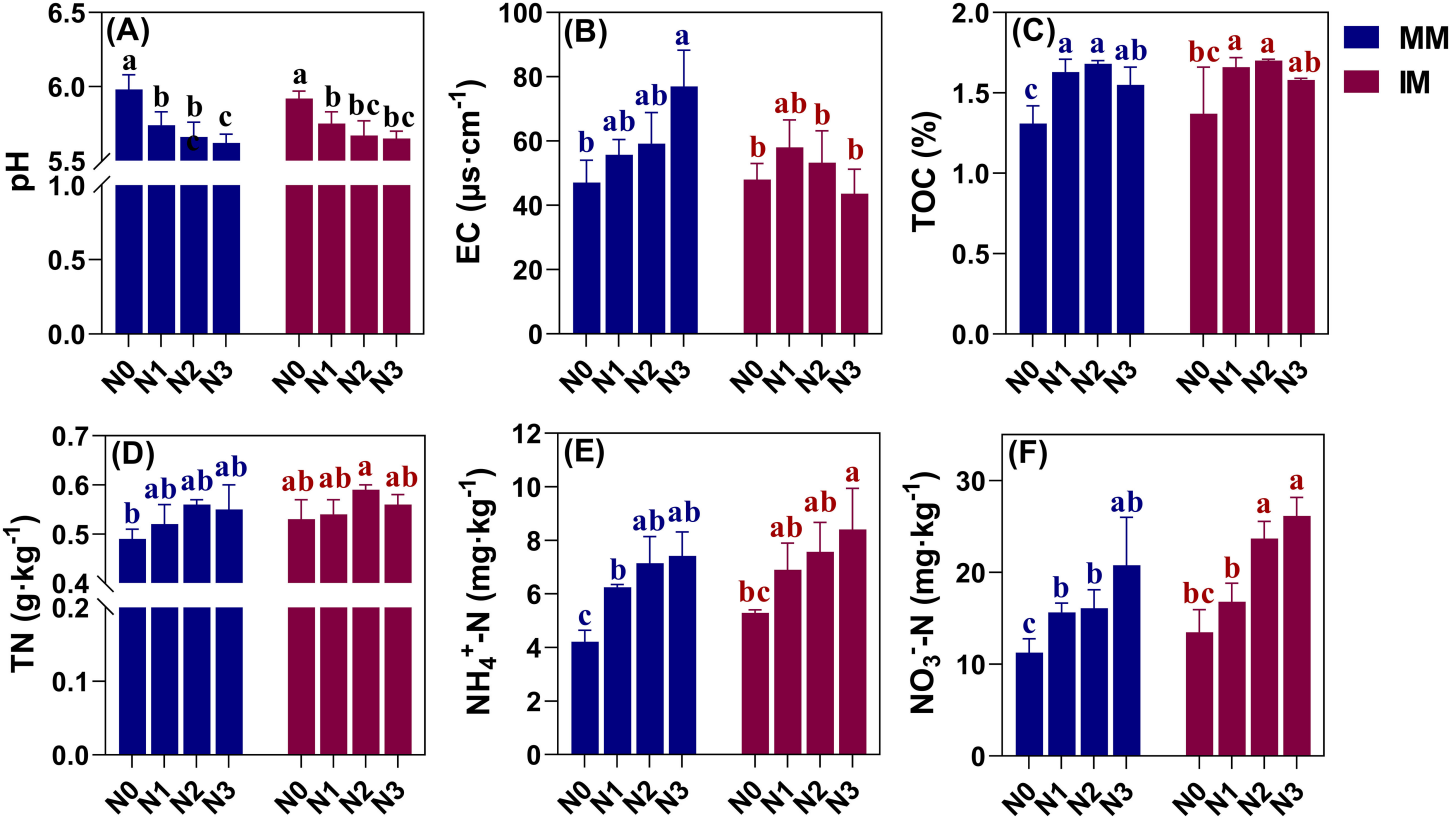
Effects of two contrasting cropping systems and nitrogen application rates on soil pH **(A)** and EC **(B)**, and the total organic carbon (TOC) **(C)**, total nitrogen (TN) **(D)**, ammonium nitrogen (NH_4_^+^-N) **(E)**, and nitrate nitrogen (NO_3_^-^-N) **(F)** in soil. Different lowercase letters (a, b, c) above the bars (mean ± SD, n = 9) indicate significant differences between treatments (P < 0.05). MM, monoculture; IM, intercropping.

Soil pH under either monoculture (MM) or intercropping (IM) tended to decrease with an increasing nitrogen application level, being lowest in the N3 treatment for both, under which it was significantly (P < 0.05) reduced by 0.36 and 0.27 vis-à-vis N0 (**Figure 2A**). However, soil pH was similar under MM and IM at the same level of nitrogen applied. Soil EC values of treatments under MM increased with greater levels of nitrogen application (**Figure 2B**), significantly rising by 63.48% in N3 over the N0 treatment (P < 0.05). However, soil EC was affected differently under IM, being highest instead at the N1 level, such that with more nitrogen applied, the soil EC values decreased, even falling below that of the N0 treatment. For example, the soil EC value of N3 was reduced by 9.17% in comparison with N0. Under MM and IM, the soil TOC content reached its maximum when the nitrogen application level was increased to N2, but adding more nitrogen led to reductions in the soil TOC content (**Figure 2C**). In this case, relative to N0, the N2 treatment significantly increased the soil TOC content by 28.24% and 24.09% (P < 0.05) but the soil TOC content did not differ significantly between MM and IM at the same nitrogen level. Overall, different nitrogen application levels emerged as the main factor driving changes in soil pH, EC and TOC, while IM could have led to weakened soil EC.

The effects of different modes of cropping and nitrogen application levels on soil nitrogen content are presented in **Figures 2D–F**. Evidently, the soil NH4^+^-N and NO_3_^–^N contents under MM and IM varied consistently, with both increasing in response to more nitrogen applied and peaking in the N3 treatment, significantly surpassing the N0 treatment by 58.98%–75.83% and 84.38%–93.93%, respectively (P < 0.05). For soil TN content, however, it was maximal at the N2 level and increasing the nitrogen application further reduced it. Notably, there was significant change in the soil TN content with increasing N application between treatments under MM or IM (P > 0.05), and only the N0 treatment under MM differed significantly from the N2 treatment under IM (P < 0.05). By comparing the different cropping systems, the outcome of a higher soil NH_4_^+^-N and NO_3_^–^N content was more pronounced under IM, increasing by 20.23% and 16.46% in response to N0 under IM vis-à-vis MM, respectively, while soil TN content increased by only 7.55%.

### 3.3 Nitrogen-use efficiency

To clarify the effects of different fertilizer application rates and cropping systems on nitrogen fertilizer use, we calculated the nitrogen-use efficiency (NUE), nitrogen agronomic efficiency (NAE), nitrogen partial productivity (NPP), and the nitrogen harvest index (**Table 3**). This analysis revealed that all indicators except NAE had the response pattern of IM > MM for a given application level, whereas NAE was always similar between the cropping systems. All indicators except NPP reached their maximum values at the N2 level, where NUE and NHI respectively increased significantly (P < 0.05) by 44.56% and 11.20% respectively, and NAE decreased by 2.67% under IM in comparison to MM. The NPP peaked at the N1 level under both IM and MM, after which it decreased significantly (P < 0.05) with more nitrogen applied. Two-way ANOVAs revealed that the application rate was the main factor influencing the nitrogen fertilizer use. Overall, the N2 level under IM had the greatest nitrogen fertilizer use.

**Table 3:** Statistics for soil indicators of nitrogen fertilizer use under two contrasting cropping systems (C) and N application rates (N).

Treatment		NUE (kgatm^-1^)	NAE (kgatm^-1^)	NPP (kgatm^-1^)	NHI (%)
MM	N0	–	–	–	77.62 ± 0.41c
	N1	17.11 ± 3.7b	26.58 ± 2.34a	76.01 ± 3.97b	79.11 ± 1.22b
	N2	20.02 ± 3.76b	26.95 ± 1.07a	64.02 ± 2.06c	81.82 ± 1.42b
	N3	17.44 ± 3.22b	17.33 ± 1.04b	46.98 ± 3.04d	80.44 ± 1.11b
IM	N0	–	–	–	86.67 ± 2.28a
	N1	28.55 ± 4.76a	23.35 ± 3.86a	95.21 ± 4.41a	85.29 ± 0.87ab
	N2	28.94 ± 1.38a	26.23 ± 1.07a	80.12 ± 1.61b	88.61 ± 0.95a
	N3	18.37 ± 1.52b	17.3 ± 3.13b	60.41 ± 2.62c	84.12 ± 1.23ab
Two-factor variance analysis (*F*-value)					
	N	**	**	**	*
	C	**	ns	**	*
	N*C	*	ns	ns	*

NUE, nitrogen-use efficiency; NAE, nitrogen agronomic efficiency; NPP, nitrogen partial productivity; NHI, nitrogen harvest index. Small letters (a, b, c) indicate significant differences between treatments (P < 0.05). Asterisks indicate a significant effect on the response variable: * P < 0.05, ** P < 0.01. MM, monoculture; IM, intercropping. ns indicate that there is no significant effect on the response variable.

### 3.4 Changes in soil microbial communities

#### 3.4.1 Alpha diversity of bacterial communities

Upon completing the sequencing in this study, the OTUs were screened for de-low content, after which the non-repetitive sequences (excluding single sequences) were clustered into OTUs based on 97% similarity, with any chimeras removed in the clustering process, to obtain representative sequences of OTUs. The final number of OTUs totaled 3601, resulting in a minimum of 30 566 optimized sequences per sample. To investigate the alpha diversity of individual soil samples, the richness (chao1 index), diversity (Shannon index and PD_whole_tree), and sequence depth (Goods_coverage) of the soil bacterial community were calculated for each cropping system and nitrogen fertilizer treatment. As seen in **Table 4**, the Goods_coverage for bacteria in all treatments was above 95%. For bacterial community diversity, the Shannon index and PD_whole_tree varied consistently among treatments, being highest at N0 level under both MM and IM modes of cropping; however, increasing the nitrogen application rate led to lower bacterial community diversity, this significantly reduced by 14.05%–17.66% and 11.73%–14.16% in N3 relative to N0 (P < 0.05). For the same level of nitrogen application, bacterial community diversity was higher under IM than MM; for example, it significantly increased by 11.86% and 10.94% (P < 0.05) under IMN0 than MMN0, but not as much (7.16%–7.88%) when more nitrogen was added up to the N3 level. In terms of bacterial community richness, the Chao1 index had an identical pattern to the diversity index, in that it was higher in IM than MM, rising by 9.13% and IMN0 compared to MMN0. Interestingly, the Chao1 index was likewise disrupted by adding more nitrogen, peaking at the N2 level in both MM and IM, with further increases in the nitrogen application significantly decreasing its value (P < 0.05). A two-way ANOVA showed that both the N application level (N) and cropping pattern (C) were highly significantly correlated (P < 0.01) with alpha diversity, and overall, intercropping increased the bacterial community’s alpha diversity regardless of N application level, with an increasing N application decreasing that diversity.

**Table 4:** Alpha diversity statistics of bacterial communities under two contrasting cropping systems (C) and four nitrogen application rates (N).

Treatment		Shannon index	Chao1 index	PD_whole_tree	Goods_coverage
MM	N0	15.59 ± 0.13bc	7718 ± 41e	519.9 ± 29.22c	0.96a
	N1	14.64 ± 0.03c	7990 ± 12d	472.0 ± 19.21d	0.96a
	N2	13.62 ± 0.03d	8306 ± 10c	467.6 ± 32.55e	0.96a
	N3	13.40 ± 0.05d	8128 ± 100d	458.9 ± 25.33e	0.96a
IM	N0	17.44 ± 0.01a	8423 ± 168c	576.8 ± 35.67a	0.96a
	N1	16.57 ± 0.01b	8700 ± 280b	546.7 ± 15.34b	0.96a
	N2	15.47 ± 0.06bc	8814 ± 22a	526.6 ± 38.53c	0.96a
	N3	14.36 ± 0.12c	8572 ± 184bc	495.1 ± 6.86cd	0.96a
Two-factor variance analysis (*F*-value)					
	N	**	**	**	ns
	C	**	**	**	ns
	N*C	*	**	**	ns

Small letters (a, b, c) indicate significant differences between treatments (P < 0.05). Asterisks indicate a significant effect on the response variable: * P < 0.05, ** P < 0.01. MM, monoculture; IM, intercropping. ns indicate that there is no significant effect on the response variable.

#### 3.4.2 Community composition for dominant bacterial genera

**Figure 3:**
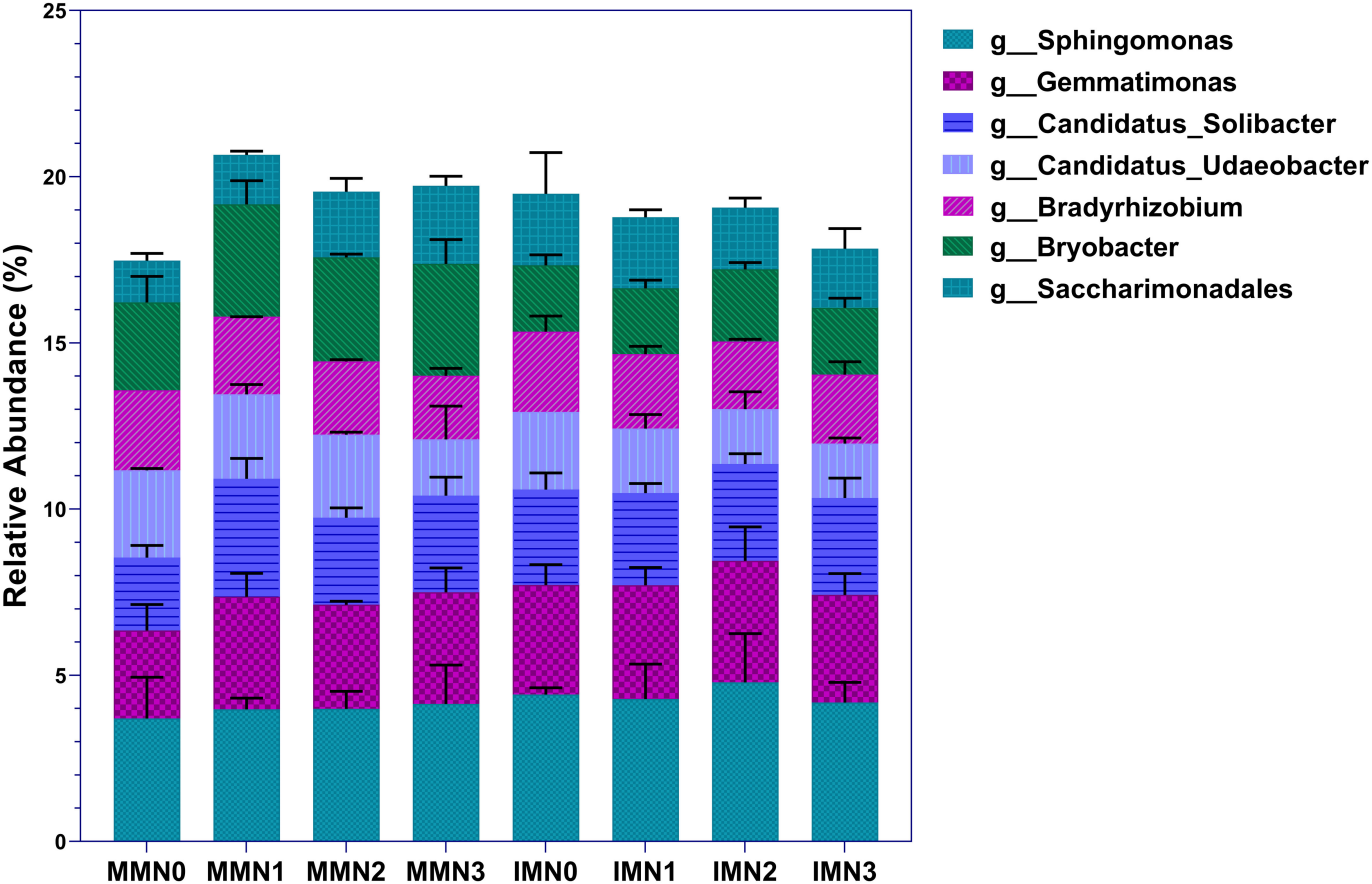
Horizontal community composition of bacterial genera in the eight treatment combinations of cropping system (IM, intercropping vs. MM, monoculture) and four levels of nitrogen fertilizer application (N1–4).

Altogether, the soil samples could be annotated to 645 bacterial genera. Of these, dominant bacterial genera accounted for 17.48%–20.66% of the total, after selecting the top-50 genera by relative abundance and excluding the seven genera whose abundance was < 1% (**Figure 3**). At N0 level, the abundance of dominant bacterial genera was 11.49% higher in IM than MM. Across the fertilizer treatment gradient, the total abundance of dominant bacterial genera was lower in IM than in MM at each N application level. This trend was driven by the enrichment of *Bryobacte* in MM at each N application level, whose relative abundance was 32.80%–70.88% higher under N0–N3 than in their counterpart IM treatments. Further comparison of the relative abundance of individual genera showed that the trends of *Sphingomonas* and *Gemmatimonas* under different modes of cropping and nitrogen application levels were consistent: IM > MM. The relative abundance of these two genera responded positively to more nitrogen applied under MM, increasing most at N1, reaching 7.50% and 27.88%, respectively. Under IM, however, their relative abundance peaked at the N2 level.

For *Candidatus_Solibacter*, the relative abundance of this genus changed the most at N0 and N1 levels, increasing by 30.84% IMN0 vis-à-vis MMN0. But in response to N1, its relative abundance increased by 61.53% compared to N0 under MM, now exceeding that of IMN1. The relative abundances of *Candidatus_Udaeobacter* and *Bryobacter* showed the same trend, namely that of MM > IM, regardless of the nitrogen application rate. However, the relative abundance of *Candidatus_Udaeobacter* decreased as the level of nitrogen application increased, with that at N3 being 35.52% and 29.67% lower under MM and IM, respectively, than in the N0 treatment. In stark contrast, *Bryobacter* abundance was maximal in the MMN1 and IMN2 treatment combinations. For *Bradyrhizobium*, its relative abundance was similar under MM and IM, as was its trend across nitrogen application rates: compared with the baseline N0 level, with more nitrogen, *Bradyrhizobium* decreased in abundance by 20.56% and 16.62% in N3 under MM and IM, respectively. The entirely opposite pattern characterized changes in *Saccharimonadales* under MM and IM. With more nitrogen added, its relative abundance under MM tended to increase, being 86.91% in the N3 than N0 treatment; however, under IM showed a decreasing trend in response to fertilization, being 17.47% lower at the N3 than N0 level. To sum up, IM increased the relative abundances of *Sphingomonas*, *Gemmatimonas*, and *Saccharimonadales* yet decreased those of *Candidatus_Udaeobacter* and *Bryobacter*, and different levels of nitrogen application had a greater impact on the enrichment of dominant bacterial genera under the MM cropping system than IM. This pointed to a superior stability of the microbial community under IM than MM.

#### 3.4.3 PCA of the bacterial community

**Figure 4:**
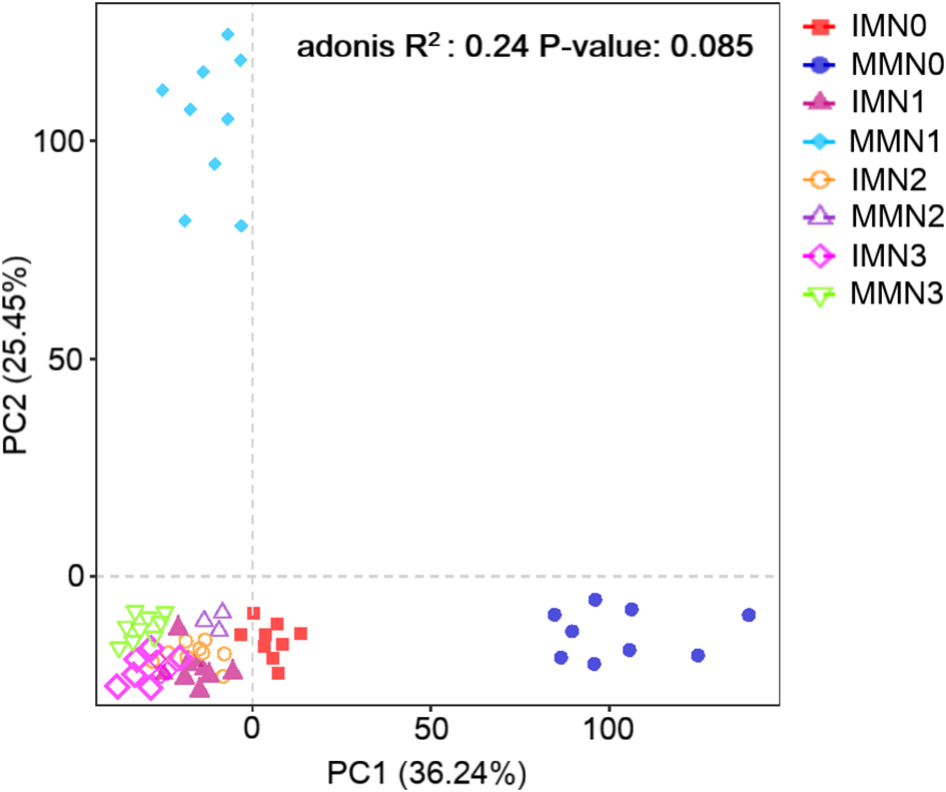
Principal component analysis (PCA) of bacterial community structure in the eight treatment combinations of cropping system (IM, intercropping vs. MM, monoculture) and four levels of nitrogen fertilizer application (N1–4).

We employed the Aitchison distance for the beta diversity analysis of soil bacterial communities. PCA was used to compare the degree of similarity existing in the diversity of the bacterial community among different samples from the eight treatment combinations (**Figure 4**). The first (PC1) and second principal component (PC2) of bacterial community structure (97% similarity) explained 36.24% and 25.45% of the total variance, respectively. There was no significant effect of differing nitrogen application rates on bacterial community structure under IM or MM (P > 0.05), and most of treatment combinations were clustered in the third quadrant (lower-left) without segregation. Unlike those, the MMN0 and MMN1 treatment combinations separated well along PC1 and PC2, respectively, indicating that their community structure differed substantially from the others (P < 0.05). Overall, the IM approach had no significant effect (P > 0.05) upon soil bacterial community structure across the nitrogen fertilizer gradient, though the differences under MM for the zero and low nitrogen application rates (N0 and N1, respectively) were significant (P < 0.05). Nonetheless, with more nitrogen applied, the soil bacterial community structure of both cropping systems closely resembled each other.

#### 3.4.4 Discrimination of differential bacterial taxa

**Figure 5:**
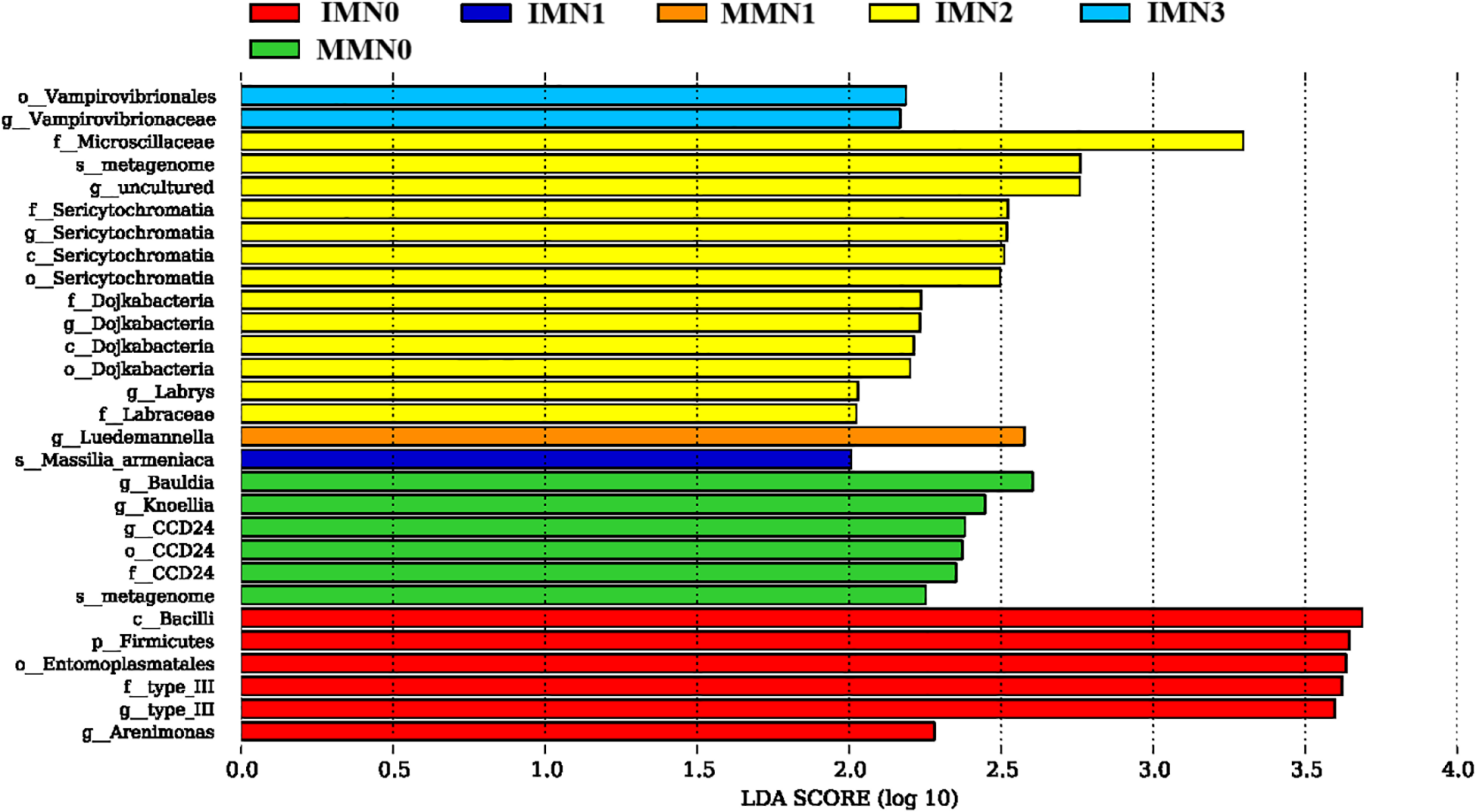
Determination of the most significantly different species in soil bacterial communities under contrasting cropping systems (IM, intercropping vs. MM, monoculture) and nitrogen application rates, using linear discriminant analysis (LDA) effect sizes (LEfSe). An LDA score ≥ 2 indicated a differential species, i.e. statistically different biomarkers. The bar length is proportional to the effect size.

It is evident that differential species mainly emerged in the IMN2, IMN0, and MMN0 treatment combinations (**Figure 5**); but not so in either MMN2 or MMN3 (thus not shown). Further analysis indicated that IMN2 harbored the most differential species, with *Microscillaceae* having the highest LDA values followed by 12 others, including *Metagenome*, *Sericytochromatia*, and *Dojkabacteria*. In the IMN0 treatment combination, the differential species consisted mainly of *Bacillus*, *Fiemicutes*, and *Entomoplasmatales* with more similar LDA values. While for MMN0, its differential species were mainly *Bauldia* and *Knoellia*. For IMN1, IMN3 and MMN1 their differential species respectively were *Vampirovibrionaceae*, *Luedemannella*, and *Massilia_armeniaca*. Altogether, the number of differential species decreased as the nitrogen application rate increased under MM, while the IM mode had the greatest number of differential species at the N2 level, indicating that the soil environment responded most to this treatment.

#### 3.4.5 Correlation analysis of dominant bacterial genera with the soil environment and yield

**Figure 6:**
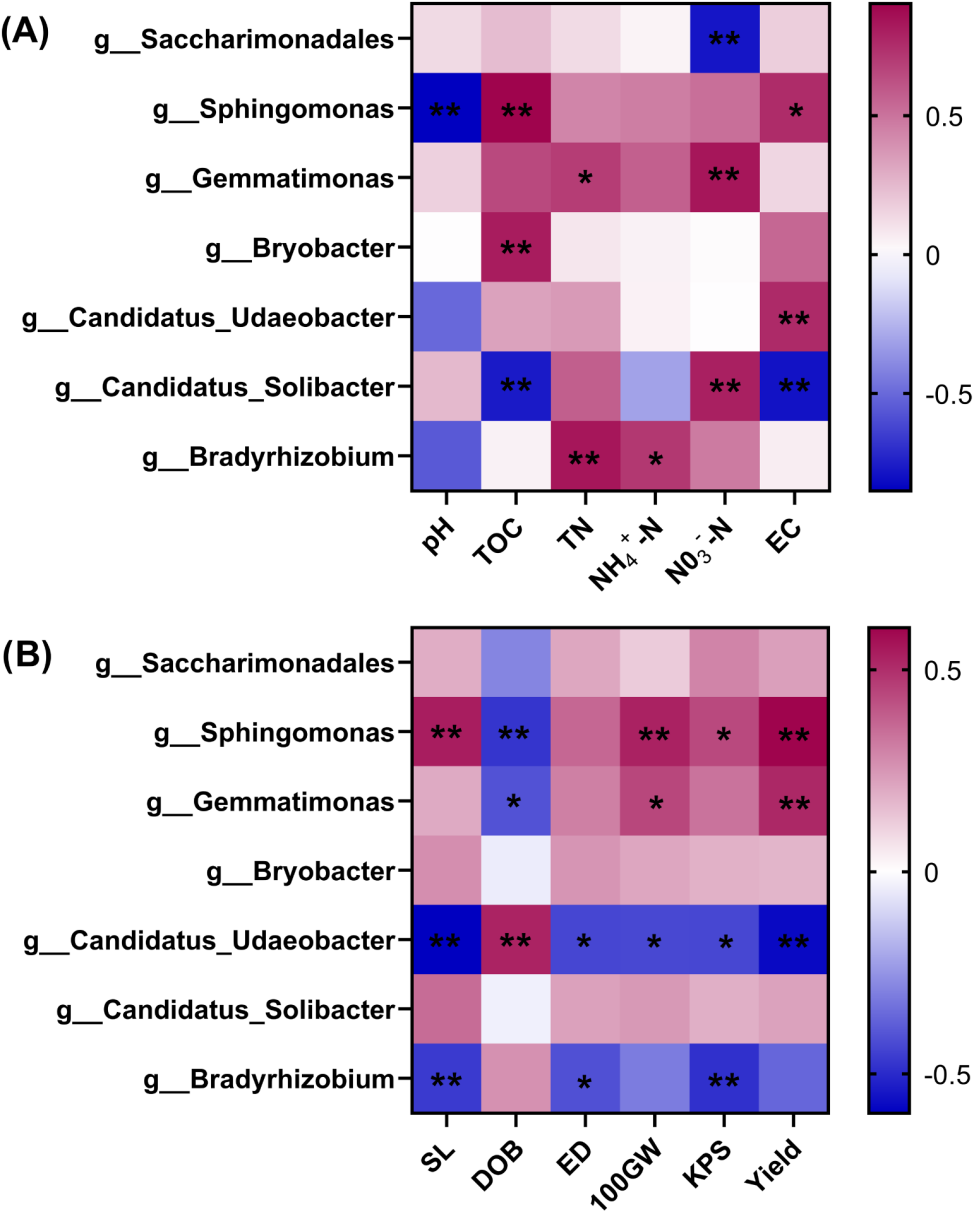
Heatmap of Pearson correlations of soil dominant bacterial genera with soil factors **(A)** and yield factors **(B)**. Red coloring indicates a positive correlation, blue coloring indicates a negative correlation; asterisks indicate a significant correlation: *P < 0.05, **P < 0.01.TOC, TN, NH_4_^+^-N and NO_3_^-^-N denote soil organic carbon, total nitrogen, ammonium nitrogen and nitrate nitrogen, respectively; SL, DOB, ED, 100GW and KPS denote maize panicle length, degree of baldness, ear diameter, 100-seed weight, and kernels in the spike, respectively; UE and CAT denote soil urease and catalase, respectively.

We summarized the dominant bacterial genera in the previous section and tallied their respective relative abundance for correlation tests with the soil environment factors and maize yield factors (**Figure 6A**). This analysis showed that *Sphingomonas* had a highly significant negative correlation with soil pH (P < 0.01) yet a positive correlation with soil TOC and EC (P < 0.05). The enrichment of *Gemmatimonas* and *Bradyrhizobium* significantly increased the soil nitrogen content; the positive correlation of *Candidatus_Udaeobacter* with soil EC was highly significant (P < 0.01), whereas *Candidatus_Solibacter* had a negative correlation (P < 0.01) with both soil TOC and EC, but a significant positive correlation with NO_3_^–^N (P < 0.01). *Bryobacter* was significantly positively correlated with the soil TOC content (P < 0.05), while *Saccharimonadales* had a highly significant negative correlation with NO_3_^—^N (P < 0.01).

In terms of yield (**Figure 6B**), enrichment of both *Sphingomonas* and *Gemmatimonas* significantly reduced the degree of baldness (DOB) (P < 0.05) and increased the spike length (SL), number of kernels in the spike (KPS), and 100-seed [grain] weight (100GW) of maize, which in turn significantly increased maize yield (P < 0.05). However, *Candidatus_Udaeobacter* and *Bradyrhizobium* had the opposite influence on yield, with enrichment of either increasing the DOB. Still, *Candidatus_Udaeobacter* displayed a greater negative influence on yield, as indicated by its relative abundance being highly significantly negatively correlated with both SL and yield (P < 0. 01) and to a lesser extent with ear thickness (ED), 100GW, and KPS (P < 0.05). Finally, a higher relative abundance of *Bradyrhizobium* significantly reduced the maize SL, ED, and KPS (P < 0.05).

#### 3.4.6 Co-occurrence network modeling of soil bacterial communities

**Figure 7:**
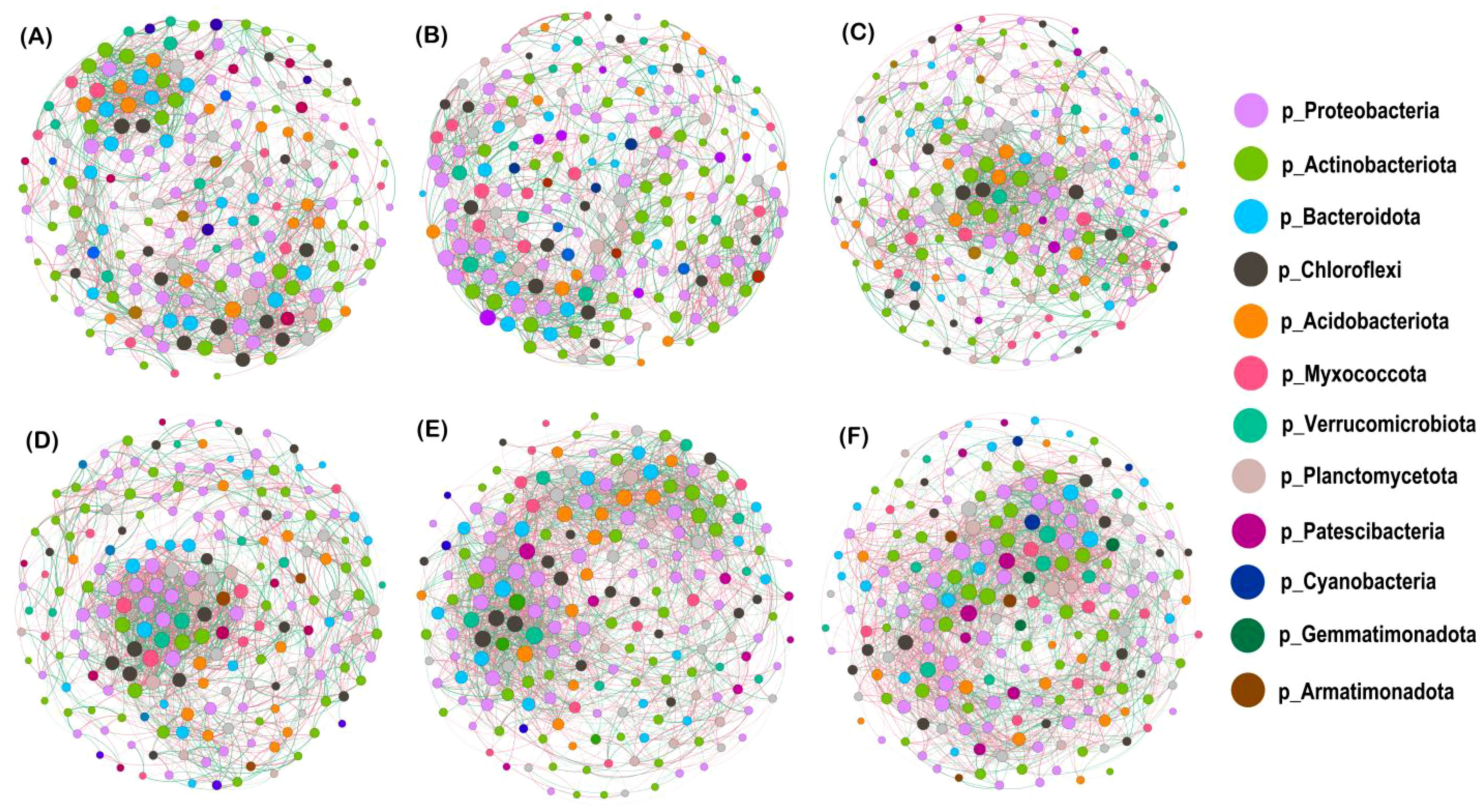
Co-occurrence network model of soil bacterial communities under different nitrogen application rates and cropping systems. **(A–D)** Different nitrogen application levels (N0, N1, N2, and N3, respectively) (MS and IS fitted by 97% similarity). **(E)** Maize monocropping and **(F)** intercropping (N0, N1, N2, and N3 fitted by 97% similarity) as contrasting cropping modes. The colored circles are different genus; the size of a circle is used to convey the average abundance of that species; line segments between circles indicate a correlation between two species, whose thickness is proportional to the correlation strengthen between them. Red lines indicate positive correlations, while green lines indicate negative correlations.

To clarify the mechanism of synergistic interaction among genera, we built co-occurrence network models of soil bacterial communities at the genus level for the top 200 abundances for different nitrogen application levels (MM and IM fitted by 97% similarity) (**Figures 7A–D**), and likewise for different cropping modes (N0–N3 fitted by 97% similarity) (**Figures 7E, F**). We also examined the topology parameters of these network models (**Table 5**) to compare the interconnections among soil bacterial communities. When compared to the N0 level, the number of soil bacterial community edges decreased by 2.30%–8.74% at the N1–N3 levels, but the proportion of positive correlations increased by 0.66%–5.76%, being highest at the N2 level, with no significant difference detected between the N3 and N0 levels; however, the number of edges and proportion of positive correlations under IM increased by 1.91% and 4.62%, respectively, vis-à-vis MM. In comparing the average degree, average weight, average clustering coefficient, and modularity, we found that all these network topology parameters were lower for the N1–N3 levels than for N0, and always higher under IM than MM. Hence, the degree of connectivity between network nodes in the IM system was likely stronger and the connections between nodes more numerous and complex.

**Table 5:** Topological properties of the soil bacterial community co-occurrence networks.

Treatment	Total nodes	Edge	Positive %	Negative%	Average degree	Average weighting	Cluster coefficient	Modularity
N0 (MM,IM)	200	1911	53.06	46.94	19.11	16.89	0.55	0.52
N1 (MM,IM)	200	1744	55.24	44.76	17.44	15.36	0.52	0.51
N2 (MM,IM)	200	1821	58.82	41.18	18.21	16.03	0.51	0.45
N3 (MM,IM)	200	1867	53.72	46.28	18.67	16.52	0.52	0.50
MM (N0-N3)	200	2197	53.71	46.29	21.97	15.55	0.43	0.40
IM (N0-N3)	200	2239	56.19	43.81	22.39	15.69	0.45	0.41

#### 3.4.7 Structural equation modeling analysis of the effects of intercropping and nitrogen application rates on maize yield

**Figure 8:**
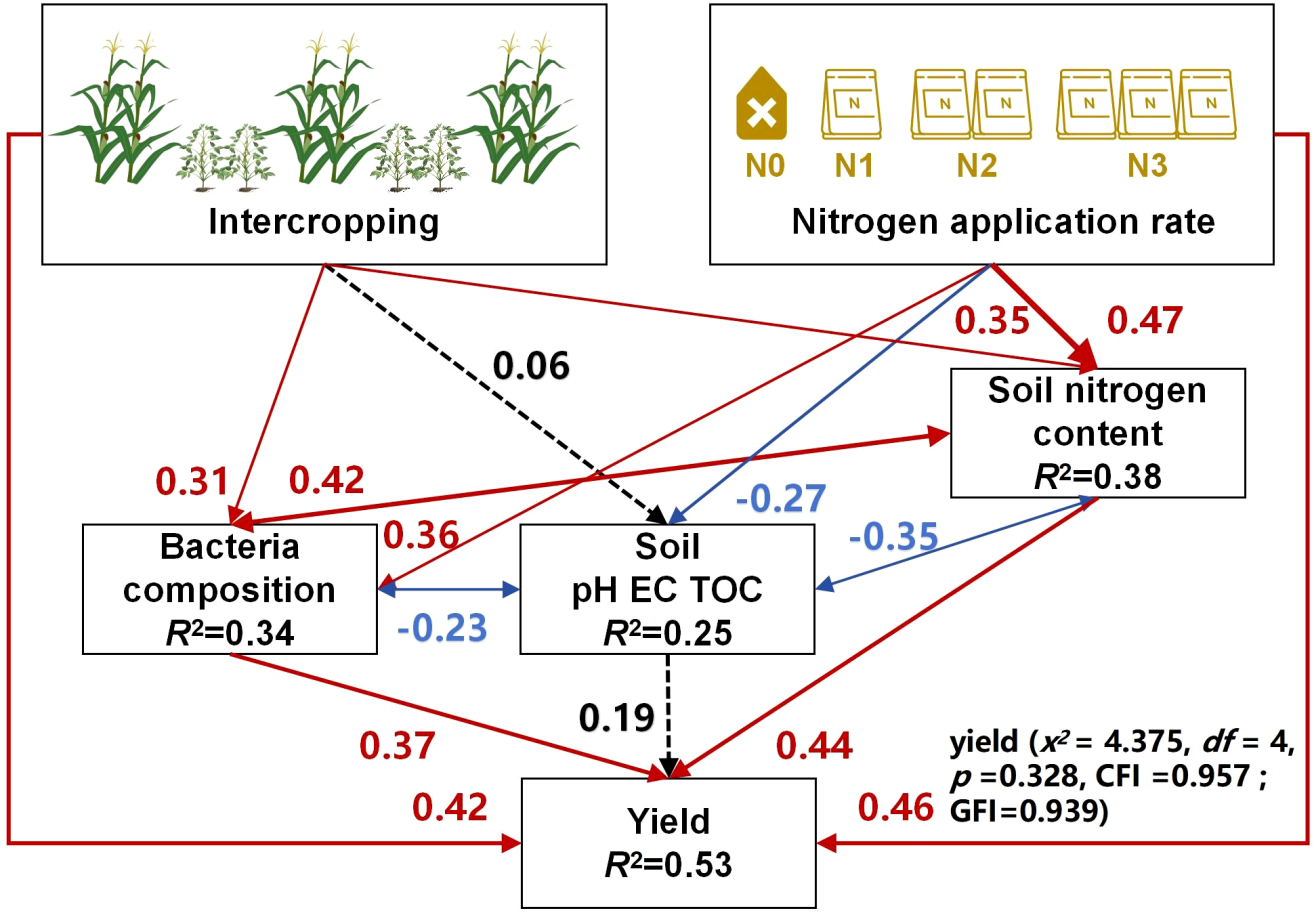
Structural equation model (SEM) describing the effects of intercropping and nitrogen application rates on soil chemical properties, bacterial colony composition, and maize yield. Blue arrows indicate negative relationships (correlations); red arrows indicate positive ones.

The SEM results (**Figure 8**) showed that both intercropping and nitrogen application could significantly increase the soil nitrogen content and also alter bacterial community composition. Soil nitrogen content and bacterial community composition were in a mutually reinforcing relationship; accordingly, the former’s increase and the latter’s enrichment could significantly reduce the soil pH, EC, and TOC content generated a synergistic effect in soil, which in turn increased the maize yield. Therefore, intercropping not only bolstered the yield but it also was critical for maintaining soil health and providing a favorable soil environment for crop growth.

## 4 Discussion

The mechanism underpinning efficient nitrogen uptake in the intercropping system between maize and legume crops has three key aspects. First is interspecific plant competition, where maize outcompetes legume crops for light and nitrogen (and other resources), which promotes the superiority of maize’s uptake of nitrogen. Second, the root system’s interaction with soil microorganisms promotes the cycling of nutrients and the transfer of nitrogen between plants, such that the nitrogen fixed by legume crops can be absorbed and utilized by maize. Third, legume crops increase tuberculation and their nitrogen-fixing gene expression is enhanced, thereby increasing their own nitrogen-fixing capacity.

### 4.1 Soil nitrogen and chemical properties

A marked increase in soil pH and salt ion concentrations in soil will lead to the degradation of crop survival/growing environment and soil nutrient deficiencies, which heavily hinders crop growth, development, and yield. In this study, however, our results show that soil pH is negligibly affected in general, being similar at each level of nitrogen application under IM or MM. This suggests intercropping does alter soil pH, whose magnitude is instead mainly influenced by the level of nitrogen application (Wang et al., 2015). Further, we find a lower soil EC in the intercropping system than monoculture (MM) but a higher soil TOC content, which can be largely attributed to the fact that IM could balance the soil nutrient use rate, effectively alleviating the inhibitory effect of soil mono-elements and enhancing soil microbial metabolism. This would have led to a greater TOC content because of the greater decomposition capacity of organic matter (Silva et al., 2022). We also found that, under intercropping, its soil TOC content was consistent with changes in its soil EC. This could be explained by the strong adsorption capacity of soil TOC, which contributes about 20%–70% to soil EC and has a certain buffering effect, delaying the movement of surface salt ions into the deeper soil, thereby augmenting EC (Wang et al., 2023b).

The nitrogen in soil is the main source of this element for plant uptake and use, and is also the primary limiting factor for high-yielding crops, so nitrogen input as fertilizer is a key practice to maintain or improve the soil nitrogen content (Knops et al., 2002). Here, the soil nitrogen supply in IM surpassed that in MM, and the improvement was especially pronounced for the NH_4_^+^-N and NO_3_^–^N contents. We attribute this benefit to the maize/soybean intercropping system, whereby the legumes are able to efficiently fix N, which is then utilized by the same crop through various transfer pathways or available in soil to subsequent non-legume crops as residues (Raza et al., 2020).

### 4.2 Rhizosphere bacterial community of maize

Soil bacteria can influence the metabolic activities of crop roots, as well as soil structure and soil fertility, to varying degrees. When crops are grown in an intercropping system, plant residues and root secretions may come into close contact with each other, directly or indirectly altering the soil bacterial community structure (Duchene et al., 2017). Compared with monocropped maize, we found a more diverse soil bacterial community in the rhizosphere soil of intercropped maize, as well as a greater relative abundance of dominant genera. This result suggests that the composition of bacterial communities in intercropped soils is heavily influenced by changes in agricultural management practices (Lai et al., 2022). However, using too much nitrogen fertilizer disrupts this trend and reduces the number of endemic differential species. This can be explained by the fact that after an excessive nitrogen application, the general plant utilization rate of the fertilizer is only 30%–50%, leaving the rest to get converted to nitrate via nitrification in the soil. That would cause soil acidification and a neutralization reaction with the inorganic carbon (carbonate) in soil, leading to the loss of inorganic carbon and eventually the full deterioration of soil, which inevitably would somehow adversely impact bacterial community diversity. Recent research has shown that increasing plant diversity to ensure the diversity of the soil microbial community can significantly improve soil fertility and reduce the number of pathogenic microorganisms (Jensen et al., 2020). Earlier, Weidner et al. (2015) found that bolstering soil bacterial diversity can improve the nitrogen nutrient supply capacity of soil, thus promoting not only plant growth but also nitrogen use; Trivedi et al. (2012) reported considerably more copies of genes involved in the ecological processes of nitrogen metabolism in healthy soils than diseased soils. This indicates that maize/soybean intercropping promotes the diversity of bacterial communities in surface soil, and thus the availability of nitrogen as well as other nutrients in arable soil. Apart from being useful in maintaining the stability of soil microbial community dynamics, it could probably help to suppress pests and diseases too, collectively promoting the growth and yield increase of intercropped crops. Among dominant bacterial genera compared between modes of cropping, our results reveal that the relative abundances of *Sphingomonas* and *Gemmatimonas* are higher under IM than MM, but this is reversed for *Bryobacter*. Other research found that, under the action of maize root secretion, Ascomycetes came to dominate, for which *Sphingomonas* and *Gemmatimonas* are the main genus members; so it was suggested this phenomenon enables the maize root system to fully utilize inorganic nutrients in soil (Zhang et al., 2018b). Arguably, subsoil intercropping conditions most likely arise through significant changes in the composition of inter-root secretions, leading to dramatic changes in bacterial colonization outcomes and population sizes of dominant bacteria. By constructing a co-occurrence network model, we learned that IM does foster an increase in the number of soil bacterial edges, an increase in the proportion of positive correlations, and an increase in species richness, in tandem with the weakening of competitive relationships between species. This is because intercropping created ecological conditions that improved the survivorship of dominant bacterial genera in surface soil, which in turn led to the enhancement of mutually beneficial relationships and a weakening of their competitive interactions (Wang et al., 2023c). This showed that IM could promote links between the genera and, when used with a reasonable nitrogen application rate (N1 or N2 level), was capable of simplifying the interrelationship between them, presumably weakening the competitive relationship between the genera and enhancing their synergistic ability.

### 4.3 Soil nutrient–microbe–yield relationships

In recent years, there has been an exponential rise in studies investigating the various effects of agricultural management practices on soil microbial communities (Zhao et al., 2022). Despite that staggering research effort, it is still difficult to elucidate general soil nutrient–microbe–yield relationships and the pathways influencing their dynamics under intercropping systems. Soil microbial community structure and functional diversity are closely tied to restored soil fertility and better soil quality (Yang et al., 2023). It is reasonable to think that plant–microbe interactions between roots are closely related to the growth and yield formation of intercrops.

Here, through a comprehensive soil nutrient–microbe–yield correlation analysis, we find that enrichment of *Gemmatimonas* and *Bradyrhizobium* could drastically increase the soil nitrogen content. Firstly, *Gemmatimonas* is a self-photosynthesizing genus rich in bacterial chlorophyll members; in addition, this genus can reduce N_2_O to nitrate in soil (Zou et al., 2023). Secondly, *Bradyrhizobium* bacteria is able to form a nitrogen-fixing symbiosis with *Rhizobium* in soybean’s root system, which in turn can increase the soil nitrogen content (Cheng et al., 2023). However, maize is a plant with high fertilizer absorption capacity and high fertilizer requirement. So, in the intercropping system, due to the difference in morphological structure between maize and soybean plants, the reduced absorption of light energy due to their asymmetric competition inhibited the growth and root development of soybean to a certain extent, which indirectly constrained its symbiotic nitrogen-fixing ability (Liu et al., 2018). If so, this would explain rather well why the genus *Bradyrhizobium* was negatively correlated with the maize yield factors in our study. On the contrary, *Saccharimonadales* has a strong negative correlation with soil NO_3_^–^N, likely because this genus has the function of denitrifying and removing phosphorus, and is mainly involved in the denitrification process leading to soil nitrogen loss (Wang et al., 2022a). We also uncovered a pronounced negative correlation of *Sphingomonas* with soil pH but its positive correlation with soil TOC and yield. The first may be explained by the role of this genus in remediating heavy metal contamination, being vital to restoring soil fertility and promoting plant growth (Liu et al., 2022). The second result can be explained by the ability of *Sphingomonas* to decompose soil carbohydrates (pentoses, hexoses, disaccharides), which produces large amounts of acid during oxidation, which then lowers the soil pH (Cuartero et al., 2022). A greater relative abundance of *Candidatus_Solibacte* was associated with declines in TOC and EC, and an increasing content of soil NO_3_^–^N. *Candidatus_Solibacte* is known to effectively decompose soil organic matter and reduce soil nitrate and nitrite by consuming the soil carbon sources, which in turn slows or prevents soil acidification (Zhang et al., 2018a). *Candidatus_Udaeobacter* can release antibiotics that cause other microbial cells to lyse and release their nutrients (Guo et al., 2024). We did find a highly significant negative correlation between this genus and maize yield, but although nutrient contents were augmented, the lysis-induced death of other microorganisms may have disrupted the structural composition of soil bacterial communities, which in turn could have affected their functioning.

### 4.4 Breakthrough areas for future research

This study’s results suggest that exploring diversified intercropping patterns that provide reciprocal benefits to different plants, by harnessing inherent differences in their ecological niches, should continue to be the core focus of future research in the intercropping industry. In this study, nitrogen uptake in the intercropping system reached its maximum when 240 kg ha^-1^ of nitrogen fertilizer was applied. Hence, an appropriate application rate of nitrogen fertilizer could effectively improve nitrogen’s uptake and accumulation in the general corn–soybean intercropping system.

Currently, most research on intercropping mechanisms tends to focus on plant growth and nutrient uptake and utilization conditions, though some studies have analyzed changes that occur in soil enzymes and their activity. The more intertwined factors, such as the soil–soil enzyme–soil microorganism interactions, under this intercropping pattern have not been studied yet, and the vast majority of published studies are restricted to the soil or soil–soil enzyme level. Therefore, research on intercropping systems should focus on the holistic analysis of each mechanistic link involved, and future studies should also try to investigate the whole intercropping system, from its environmental factors to cultivated plants, down to its soil microorganisms. The more comprehensive the research done, the more conducive its knowledge gained is to the discovery of intercropping mechanisms, and for promoting the development of the intercropping industry. Finally, the use of high-throughput sequencing technology offers a fast and convenient way to analyze intercropping soils and to explore how the soil microbial community influences plant growth/yield changes, leading to a richer, more accurate understanding of intercropping systems and their functioning.

## 5 Conclusion

This study’s results show that under a maize/soybean intercropping pattern, the N2 level (240 kg ha^-1^) was best suited to achieve a high maize yield, for which the nitrogen-use efficiency was also maximized. We find that the maize crop yield is also correlated with the dominant soil microbial genera and soil nitrogen content. The main ways by which an intercropping mode increases yield, as well as the gradient changes under different N application levels, are also clarified. In the context of research on promoting crop yields and the efficient use of nitrogen fertilizer in intensive agriculture, we believe that intercropping’s advantages are more pronounced when an appropriate amount of nitrogen is applied, since using too much can render moot these benefits.

## Data Availability

The original contributions presented in the study are included in the article/supplementary material. Further inquiries can be directed to the corresponding authors.
